# Mutation Rate Inferred From Synonymous Substitutions in a Long-Term Evolution Experiment With *Escherichia coli*

**DOI:** 10.1534/g3.111.000406

**Published:** 2011-08-01

**Authors:** Sébastien Wielgoss, Jeffrey E. Barrick, Olivier Tenaillon, Stéphane Cruveiller, Béatrice Chane-Woon-Ming, Claudine Médigue, Richard E. Lenski, Dominique Schneider

**Affiliations:** * Laboratoire Adaptation et Pathogénie des Micro-Organismes (LAPM), Université Joseph Fourier Grenoble 1, 38042 Grenoble Cedex 9, France; † Centre National de la Recherche Scientifique, Unité Mixte de Recherche (CNRS UMR) 5163, 38042 Grenoble Cedex 9, France; ‡ Chemistry and Biochemistry, Institute for Cell and Molecular Biology, University of Texas at Austin, Austin, TX 78712; § Institut National de la Santé et de la Recherche Médicale, Unité Mixte de Recherche (INSERM, UMR) S 722, 75018 Paris, France; ** Université Paris Diderot, Sorbonne Paris Cité, UMR-S 722, Faculté de Médecine, Site Xavier Bichat, 75018 Paris, France; †† CNRS-UMR 8030 and Commissariat à l'Energie Atomique CEA/DSV/IG/Genoscope LABGeM, 91057 Evry Cedex, France; ‡‡ Microbiology and Molecular Genetics, Michigan State University, East Lansing, Michigan 48824

**Keywords:** genomic base-substitution rate, experimental evolution, molecular evolution, mutation pressure, next-generation sequencing

## Abstract

The quantification of spontaneous mutation rates is crucial for a mechanistic understanding of the evolutionary process. In bacteria, traditional estimates using experimental or comparative genetic methods are prone to statistical uncertainty and consequently estimates vary by over one order of magnitude. With the advent of next-generation sequencing, more accurate estimates are now possible. We sequenced 19 *Escherichia coli* genomes from a 40,000-generation evolution experiment and directly inferred the point-mutation rate based on the accumulation of synonymous substitutions. The resulting estimate was 8.9 × 10^−11^ per base-pair per generation, and there was a significant bias toward increased AT-content. We also compared our results with published genome sequence datasets for other bacterial evolution experiments. Given the power of our approach, our estimate represents the most accurate measure of bacterial base-substitution rates available to date.

Mutations and genetic recombination provide the variation that fuels adaptation. Knowledge of mutation rates is therefore an important component of a quantitative evolutionary theory (Lynch 2010). In bacteria, spontaneous base-substitution rates have been estimated by Luria-Delbrück fluctuation tests using selective conditions (Drake 1991; Lynch 2006, 2010 and references therein) and by comparing DNA sequences from lineages with approximately known divergence times (Ochman *et al.* 1999). Both methods have limitations. The former requires knowledge of the mutational target size for the relevant phenotype and makes assumptions concerning growth and selection that do not always hold in practice (Sniegowski and Lenski 1995). The latter assumes that synonymous substitutions are selectively neutral, requires estimates of generation times in nature, and is subject to additional uncertainty when there is recombination or selection on codon usage and GC-content (Balbi *et al.* 2009; Sharp *et al.* 2010; Touchon *et al.* 2009). Given these uncertainties, it is not surprising that the mutation rates estimated for *E. coli* using these two approaches differ by more than an order of magnitude (Drake 1991; Ochman *et al.* 1999).

More direct measurements of mutation rates are now possible using whole-genome sequences of isolates sampled from evolution experiments. We have previously applied this approach to one population from the long-term evolution experiment with *E. coli* (Barrick *et al.* 2009; Barrick and Lenski 2009) in which 12 populations have been propagated independently for over 40,000 generations (Lenski 2004; Philippe *et al.* 2007). Here, we resequenced genomes of 19 clones that were sampled from 8 populations (Table 1 and supporting information, Table S1) that did not evolve elevated mutation rates early in the experiment (Cooper and Lenski 2000; Sniegowski *et al.* 1997).

**Table 1:** Description of 35 synonymous mutations observed in 19 genomes sampled from eight evolving populations

Population	Genome Position*^a^*	Gene	Base Change	Sequenced Clones*^b^*
Ara–1	–	–	–	20K-A, 20K-B, 20K-C
Ara–3	756,799	*tolR*	C→T	30K-B, 40K
	2,613,609	*purL*	G→A	30K-B
	2,642,843	*yfiQ*	G→T	30K-B
	2,983,794	*yggW*	C→T	40K
	3,141,566	*ygjE*	C→T	40K
	3,407,922	*kefB*	C→A	40K
	4,111,342	*metL*	C→T	30K-A
	4,177,963	*hemE*	T→G	30K-A
	4,107,018	*ECB_03822*	T→A	30K-B, 40K
	4,313,510	*eptA*	C→T	40K
Ara–5	157,626	*htrE*	A→T	40K-B
	307,594	*yahC*	C→T	40K-A, 40K-B, 40K-C
	3,107,610	*ygiN*	T→A	40K-A, 40K-B, 40K-C
Ara–6	857,058	*moeB*	C→T	40K-B
	1,352,030	*sapC*	G→T	40K-B
	2,087,738	*mdtA*	C→A	40K-A, 40K-B
	2,095,621	*mdtD*	G→A	40K-A
	3,482,212	*malT*	G→A	40K-B
Ara+1	132,062	*lpd*	C→T	40K-A
	239,002	*dnaQ*	A→C	40K-B
	3,124,208	*yqiI*	G→A	40K-A
	3,308,106	*yhcB*	G→A	40K-A, 40K-B
	3,409,316	*yheS*	T→G	40K-A, 40K-B
	3,527,027	*livH*	C→A	40K-B
	3,910,606	*yifB*	T→G	40K-B
	4,133,104	*ppc*	G→A	40K-A, 40K-B
Ara+2	1,083,668	*wrbA*	C→T	40K-A
	–	–	–	40K-B
Ara+4	420,328	*cyoB*	A→C	40K-A, 40K-B
	2,772,320	*Iap*	A→C	40K-A, 40K-B
	3,061,109	*ECB_02854*	G→A	40K-A
Ara+5	122,591	*ampE*	T→A	40K-A, 40K-B
	212,865	*ldcC*	T→C	40K-A, 40K-B
	1,317,194	*trpC*	G→A	40K-A, 40K-B
	2,009,188	*yoeF*	G→T	40K-A, 40K-B
	2,251,393	*napA*	G→A	40K-A, 40K-B

^a^ Genome position in the ancestral reference strain REL606 [GenBank:NC_012967.1].

^b^ 20K, 30K, and 40K indicate clones sampled after 20,000, 30,000, and 40,000 generations, respectively, and labels A, B and C indicate different clones from the same generation.

## Materials and Methods

### Mutation identification

Genomes were resequenced on the Illumina Genome Analyzer platform using one lane of single-end 36-bp reads per genome. Candidate point mutations were identified in comparison to the ancestral genome of REL606 [GenBank:NC_012967.1] using three computational approaches: (i) the *SNiPer* pipeline (Marchetti *et al.* 2010); (ii) the *breseq* pipeline (Barrick *et al.* 2009, freely available online at http://barricklab.org/breseq); and (iii) an unpublished algorithm (O. Tenaillon). All candidates were then examined manually to account for local misalignment errors relative to the reference genome that resulted from gene conversion events, mobile element insertions, and large insertions and deletions. Table S1 presents the resulting consensus list of all synonymous substitutions arranged by population and clone. The dN/dS ratios were calculated for each clone according to Comeron (1995) as implemented in the *libsequence* library (Thornton 2003).

### Synonymous target site calculations

For whole-genome studies of mutations in bacterial evolution experiments, we used in-house scripts to calculate the exact number of protein-coding sites in the ancestral genome according to gene annotations. The effective number of synonymous target sites was approximated as one-third of this number, as three mutational changes are possible from any ancestral base. This analysis does not take into account base composition effects or the small changes in genome size during these experiments. The sequence records used for other published studies were downloaded from Genbank (Accessions: NC_000913.2, AC_000091.1, NC_008095.1, and NC_003197.1). For our dataset, we used the Genbank sequence record for *E. coli* B strain REL606 (Accession: NC_012967.1) with updated gene annotations. Data files and Perl scripts for performing this analysis are available on J.E.B.’s web site (http://barricklab.org/amr).

### Mutation rate estimate

We used a maximum-likelihood approach to estimate the rates of all six possible types of base-pair substitution mutations. This approach assumed that synonymous substitutions of a given type accumulated as a Poisson process with an expected number equal to the mutation rate multiplied by the number of generations elapsed and the total number of genomic sites at risk for synonymous substitutions of that type. This last factor corrected for regions of the ancestral genome where mutations could not be called in an evolved genome due to deletions, low coverage, or repetitive sequences, as output by the *breseq* pipeline.

We corrected for pseudo-replication due to shared evolutionary history by averaging the calculated log likelihoods for genomes within population blocks. The overall point-mutation rate was then calculated by weighting the separately estimated rates for each type of mutation by the frequency of corresponding sites in the ancestral genome. Tukey’s jackknife method was used to estimate overall confidence limits from the statistics of resampled (delete–1) datasets that each dropped all genomes from a single population. Data files and Perl and R scripts for performing this analysis are available on J.E.B.’s web site (http://barricklab.org/amr).

## Results and Discussion

We analyzed synonymous substitutions because, when examining all mutations in the 19 clones, we found dN/dS ratios higher than 1.0 for all but one (Table S1). This observation supports pervasive ongoing positive selection through 40,000 generations in these experimental populations (Barrick *et al.* 2009). Therefore, non-synonymous mutations are inappropriate for estimating the point-mutation rate.

From population genetics theory, the expected number of synonymous mutations in an evolved clone relative to its ancestor is equal to the product of the intrinsic base-substitution rate, the number of genomic sites at risk for synonymous mutations, and the number of elapsed generations (Kimura 1983). The only requisite assumption is that most synonymous mutations are selectively neutral. Importantly, the expected rate of accumulation of neutral mutations in the lineage leading to any particular clone is not affected by selection at other sites in the genome, because an asexual lineage simply represents a chain of replication events spanning the specified number of generations (Barrick *et al.* 2009; Kimura 1983).

We observed a total of 52 synonymous substitutions in the 19 resequenced genomes (Table S1). However, multiple genomes sampled from the same population are not independent because they share some portion of their history; thus, there were only 35 mutational events (Table 1). We used a resampling procedure to account for this pseudo-replication of multiple genomes isolated from a single population (see supporting information). The resulting estimate of the point-mutation rate is 8.9 × 10^−11^ per bp per generation (Tukey’s jackknife 95% confidence interval, 4.0–14 × 10^−11^ per bp per generation). This estimate corresponds to a total genomic rate of 0.00041 per generation given the ancestral genome size of 4.6 × 10^6^ bp.

Our inferred point-mutation rate is intermediate to other previous estimates based on experimental (Drake 1991) and comparative methods (Ochman *et al.* 1999). These earlier studies yielded estimates of 5.4 × 10^−10^ per bp per generation and 1.5 to 4.5 × 10^−11^ per bp per generation, respectively. Given the limitations of these approaches as noted above, our estimate is probably more accurate. This greater accuracy derives from the accumulation of mutational events across 300,000 generations (summed over the eight replicate populations) and over the entire genome, coupled with precise knowledge of the number of elapsed generations and the reasonable presumption of selective neutrality or near-neutrality for most synonymous mutations. At the same time, it must also be emphasized that mutation rates may differ between strains and species, and they may change depending on the environmental conditions experienced by the cells (Bjedov *et al.* 2003).

To put our estimate into context, we performed a similar analysis of all other published whole-genome datasets for bacterial evolution experiments with known numbers of generations (Table 2). Taking the other experiments together, we found 10 synonymous SNPs in 18 independently evolved (nonmutator) clones in a total of 30,550 generations. These other datasets combined thus provide only ∼10% of the power, in terms of cumulative generations, as the long-term dataset that we have generated and analyzed. As a consequence, the estimated point-mutation rates for these other experimental systems are subject to much greater statistical uncertainty.

**Table 2:** Base-substitution rates estimated from evolution experiments with whole-genome data

Study	Bacterial Strain	Clones	Cumulative Generations	Synonymous Sites (bp)*^a^*	Synonymous Mutations	*µ* × 10^−11^ (per bp per generation)*^b^*
This study	*Escherichia coli* B REL606	19	300,000	941,000	25 (52)*^c^*	8.9 [5.7–13]
Conrad *et al.* (2009)Lee and Palsson (2010)	*Escherichia coli* K-12 MG1665	12	10,700	930,000	5	50 [16–120]

Kishimoto *et al.* (2010)	*Escherichia coli* W3110	4	13,850	945,000	2	15 [1.9–55]
Lind and Andersson (2008)	*Salmonella typhimurium* LT2	1	5000	990,000	2	40 [4.9–150]
Velicer *et al.* (2006)	*Myxococcus xanthus* DK1622	1	1000	2,140,000	1	47 [1.2–260]

For these calculations, we used only independently evolved end-point clones, and we pooled data from replicate lineages started from the same ancestral strain.

^a^ The effective synonymous target size was calculated from the ancestral genome sequences (see *Materials and Methods*).

^b^ The mutation rate *µ* (per bp per generation) was calculated as the number of observed synonymous mutations divided by the product of the total number of generations and the effective number of synonymous target sites. Brackets indicate 95% confidence limits estimated from a binomial distribution. These estimates do not take into account base composition or changes in genome size.

^c^ For comparison with the other datasets, we used only the first clone sequenced at the latest nonmutator time point from each of the eight long-term populations: 20K-A for Ara-1,40K for Ara-3, and 40K-A for the other six populations (Table 1). There were 25 synonymous mutations in these clones and 52 overall in the dataset. A more accurate estimate of *µ* and its uncertainty for the long-term lines takes into account the multiple clones sequenced from the same population, the pseudo-replication of clones from the same population, the base signatures of the mutations, and changes in genome size. That comprehensive analysis yields 8.9 [4.0–14] × 10^−11^ per bp per generation (see text).

**Figure 1:**
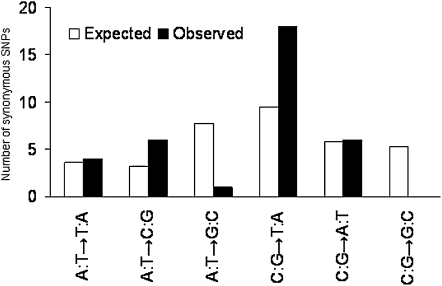
Expected and observed mutational spectra for synonymous point mutations. White and black bars show the expected and observed base-pair changes, respectively. The expected values reflect the actual base-pair frequencies in the genome and the probability that a particular base-pair mutation (*e.g.*, from C:G to T:A) produces a synonymous change.

With 35 independent synonymous mutations, we were also able to examine the mutational spectrum of base substitutions (Figure 1). After correcting for the sequence composition of genomic sites at risk for synonymous mutations in the ancestral genome, the observed transition-to-transversion ratio of 1:1.99 did not differ significantly from the 1:2 ratio expected if there were a uniform probability of all six base-substitution mutations (two-tailed binomial test, *P* = 0.61). However, transitions were highly skewed. Mutations from C:G to T:A were 14.5 times as likely as A:T to G:C mutations after accounting for sequence composition (two-tailed binomial test, *P* = 0.00027). This finding is consistent with other recent studies that found a strong mutational bias toward increased AT composition in bacteria (Balbi *et al.* 2009; Hershberg and Petrov 2010; Hildebrand *et al.* 2010). This bias in mutation pressure explains the pattern of synonymous mutations seen in our study, and it also implies that selection or gene conversion must account for the characteristic GC-contents observed in divergent groups of bacteria over much longer evolutionary timescales (Rocha and Feil 2010).

## Supplementary Material

Supporting Information

## References

[bib1] Balbi K. J. Rocha E. P. Feil E. J., 2009 The temporal dynamics of slightly deleterious mutations in *Escherichia coli* and *Shigella spp*. Mol. Biol. Evol. 26: 345–355 18984902 10.1093/molbev/msn252

[bib2] Barrick J. E. Lenski R. E., 2009 Genome-wide mutational diversity in an evolving population of *Escherichia coli*. Cold Spring Harb. Symp. Quant. Biol. 74: 119–129 19776167 10.1101/sqb.2009.74.018 PMC2890043

[bib3] Barrick J. E. Yu D. S. Yoon S. H. Jeong H. Oh T. K., 2009 Genome evolution and adaptation in a long-term experiment with *Escherichia coli*. Nature 461: 1243–1247 19838166 10.1038/nature08480

[bib4] Bjedov I. Tenaillon O. Gérard B. Souza V. Denamur E., 2003 Stress-induced mutagenesis in bacteria. Science 300: 1404–1409 12775833 10.1126/science.1082240

[bib5] Comeron J. M., 1995 A method for estimating the numbers of synonymous and nonsynonymous substitutions per site. J. Mol. Evol. 41: 1152–1159 8587111 10.1007/BF00173196

[bib6] Conrad T. Joyce A. Applebee M. K. Barrett C. Xie B., 2009 Whole-genome resequencing of *Escherichia coli* K-12 MG1655 undergoing short-term laboratory evolution in lactate minimal media reveals flexible selection of adaptive mutations. Genome Biol. 10: R118 19849850 10.1186/gb-2009-10-10-r118 PMC2784333

[bib7] Cooper V. S. Lenski R. E., 2000 The population genetics of ecological specialization in evolving *E. coli* populations. Nature 407: 736–739 11048718 10.1038/35037572

[bib8] Drake J. W., 1991 A constant rate of spontaneous mutation in DNA-based microbes. Proc. Natl. Acad. Sci. U S A 88: 7160–7164 1831267 10.1073/pnas.88.16.7160 PMC52253

[bib9] Hershberg R. Petrov D. A., 2010 Evidence that mutation is universally biased towards AT in bacteria. PLoS Genet. 6: e1001115 20838599 10.1371/journal.pgen.1001115 PMC2936535

[bib10] Hildebrand F. Meyer A. Eyre-Walker A., 2010 Evidence of selection upon genomic GC-content in bacteria. PLoS Genet. 6: e1001107 20838593 10.1371/journal.pgen.1001107 PMC2936529

[bib11] Kimura M., 1983 The Neutral Theory of Molecular Evolution. Cambridge University Press, Cambridge, UK

[bib12] Kishimoto T. Iijima L. Tatsumi M. Ono N. Oyake A., 2010 Transition from positive to neutral in mutation fixation along with continuing rising fitness in thermal adaptive evolution. PLoS Genet. 6: e1001164 20975944 10.1371/journal.pgen.1001164 PMC2958811

[bib13] Lee D.-H. Palsson B. O., 2010 Adaptive evolution of *Escherichia coli* K-12 MG1655 during growth on a nonnative carbon source, L-1,2-Propanediol. Appl. Environ. Microbiol. 76: 4158–4168 20435762 10.1128/AEM.00373-10 PMC2897412

[bib14] Lenski R. E., 2004 Phenotypic and genomic evolution during a 20,000-generation experiment with the bacterium *Escherichia coli*. Plant Breed. Rev. 24: 225–265

[bib15] Lind P. A. Andersson D. I., 2008 Whole-genome mutational biases in bacteria. Proc. Natl. Acad. Sci. U S A 105: 17878–17883 19001264 10.1073/pnas.0804445105 PMC2584707

[bib16] Lynch M., 2006 The origins of eukaryotic gene structure. Mol. Biol. Evol. 23: 450–468 16280547 10.1093/molbev/msj050

[bib17] Lynch M., 2010 Evolution of the mutation rate. Trends Genet. 26: 345–352 20594608 10.1016/j.tig.2010.05.003 PMC2910838

[bib18] Marchetti M. Capela D. Glew M. Cruveiller S. Chane-Woon-Ming B., 2010 Experimental evolution of a plant pathogen into a legume symbiont. PLoS Biol. 8: e1000280 20084095 10.1371/journal.pbio.1000280 PMC2796954

[bib19] Ochman H. Elwyn S. Moran N. A., 1999 Calibrating bacterial evolution. Proc. Natl. Acad. Sci. U S A 96: 12638–12643 10535975 10.1073/pnas.96.22.12638 PMC23026

[bib20] Philippe N. Crozat E. Lenski R. E. Schneider D., 2007 Evolution of global regulatory networks during a long-term experiment with *Escherichia coli*. Bioessays 29: 846–860 17691099 10.1002/bies.20629

[bib21] Rocha E. P. C. Feil E. J., 2010 Mutational patterns cannot explain genome composition: are there any neutral sites in the genomes of bacteria? PLoS Genet. 6: e11 10.1371/journal.pgen.1001104 PMC2936526 20838590

[bib22] Sharp P. M. Emery L. R. Zeng K., 2010 Forces that influence the evolution of codon bias. Philos. Trans. R. Soc. Lond. B Biol. Sci. 365: 1203–1212 20308095 10.1098/rstb.2009.0305 PMC2871821

[bib23] Sniegowski P. D. Lenski R. E., 1995 Mutation and adaptation: the directed mutation controversy in evolutionary perspective. Annu. Rev. Ecol. Syst. 26: 553–578

[bib24] Sniegowski P. D. Gerrish P. J. Lenski R. E., 1997 Evolution of high mutation rates in experimental populations of *Escherichia coli*. Nature 387: 703–705 9192894 10.1038/42701

[bib25] Thornton K., 2003 Libsequence: a C++ class library for evolutionary genetic analysis. Bioinformatics 19: 2325–2327 14630667 10.1093/bioinformatics/btg316

[bib26] Touchon M. Hoede C. Tenaillon O. Barbe V. Baeriswyl S., 2009 Organised genome dynamics in the *Escherichia coli* species results in highly diverse adaptive paths. PLoS Genet. 5: e1000344 19165319 10.1371/journal.pgen.1000344 PMC2617782

[bib27] Velicer G. J. Raddatz G. Keller H. Deiss S. Lanz C., 2006 Comprehensive mutation identification in an evolved bacterial cooperator and its cheating ancestor. Proc. Natl. Acad. Sci. U S A 103: 8107–8112 16707573 10.1073/pnas.0510740103 PMC1472437

