# Supplementary material for: Mutation Rate Inferred From Synonymous Substitutions in a Long-Term Evolution Experiment With Escherichia coli
Source: G3 (Bethesda). 2011 Aug 1;1(3):183–6. doi: 10.1534/g3.111.000406 (PMC3246271; doi:10.1534/g3.111.000406)
Supplement: Supporting Information [file supp_1_3_183__index.html]

Supporting Information 

# Mutation Rate Inferred From Synonymous Substitutions in a Long-Term Evolution Experiment With *Escherichia coli*

## Supporting Information for Wielgoss *et al.*, 2011

**Files in this Data Supplement:**

- Table S1 - List  of  synonymous  base‐substitutions  in  the  sequenced  genomes  (PDF, 92 KB)
