# Supplementary material for: Mutation Rate Inferred From Synonymous Substitutions in a Long-Term Evolution Experiment With Escherichia coli
Source: G3 (Bethesda). 2011 Aug 1;1(3):183–6. doi: 10.1534/g3.111.000406 (PMC3246271; doi:10.1534/g3.111.000406)
Supplement: Supporting Information [file supp_1.3.183_TableS1.pdf]

TABLE S1

List of synonymous base-substitutions in the sequenced genomes

Population	Clone Label	Clone Name	Generation	Genome Size [bp]	Synonymous Sites [bp]	Synonymous Mutations	dN/dS[a]	Genome Position[b]	Gene Name	Base Change	Average Coverage [per bp]	Sequencing Facility[c]
Ancestor	-	REL606	0	4,629,812	941,021	-	-	-	-	-	-	KRIBB[d]
Ara-1	20K-A	REL8593A	20,000	4,485,227	919,303	0	Inf.	-	-	-	56.1	KRIBB[e]
Ara-1	20K-B	REL8593B	20,000	4,483,083	918,965	0	Inf.	-	-	-	36.3	Genoscope
Ara-1	20K-C	REL8593C	20,000	4,484,924	919,293	0	Inf.	-	-	-	44.9	Genoscope
Ara-3	30K-A	ZDB16	30,000	4,490,751	921,314	2	2.62	4,111,342	*metL*	C→T	107.0	MSU
								4,177,963	*hemE*	T→G		
Ara-3	30K-B	ZDB357	30,000	4,464,009	915,982	4	1.61	756,799	*tolR*	C→T	101.7	MSU
								2,613,609	*purL*	G→A		
								2,642,843	*yfiQ*	G→T		
								4,107,018	*ECB_03822*	T→A		
Ara-3	40K	REL10988	40,000	4,425,016	908,843	6	1.43	756,799	*tolR*	C→T	31.3	MSU
								2,983,794	*yggW*	C→T		
								3,141,566	*ygjE*	C→T		
								3,407,922	*kefB*	C→A		
								4,107,018	*ECB_03822*	T→A		
								4,313,510	*eptA*	C→T		
Ara-5	40K-A	REL10947	40,000	4,458,388	914,306	2	2.01	307,594	*yahC*	C→T	61.2	MSU
								3,107,610	*ygiN*	T→A		
Ara-5	40K-B	REL10948	40,000	4,458,503	914,442	3	1.41	157,626	*htrE*	A→T	205.6	MSU

								307,594	*yahC*	C→T		
								3,107,610	*ygiN*	T→A		
Ara-5	40K-C	REL10949	40,000	4,459,592	914,639	2	2.09	307,594	*yahC*	C→T	222.1	MSU
								3,107,610	*ygiN*	T→A		
Ara-6	40K-A	REL11005	40,000	4,426,313	908,744	2	3.91	2,087,738	*mdtA*	C→A	97.9	Genoscope
								2,095,621	*mdtD*	G(A		
Ara-6	40K-B	REL11006	40,000	4,471,467	916,444	4	2.01	857,058	*moeB*	C→T	121.4	Genoscope
								1,352,030	*sapC*	G→T		
								2,087,738	*mdtA*	C→ A		
								3,482,212	*malT*	G→A		
Ara+1	40K-A	REL11008	40,000	4,361,096	894,621	5	1.07	132,062	*lpd*	C→T	38.6	Genoscope
								3,124,208	*yqiI*	G→A		
								3,308,106	*yhcB*	G→A		
								3,409,316	*yheS*	T→G		
								4,133,104	*ppc*	G→A		
Ara+1	40K-B	REL11009	40,000	4,386,876	900,352	6	0.828	239,002	*dnaQ*	A→C	42.9	Genoscope
								3,308,106	*yhcB*	G→A		
								3,409,316	*yheS*	T→G		
								3,527,027	*livH*	C→A		
								3,910,606	*yifB*	T→G		
								4,133,104	*ppc*	G→A		
Ara+2	40K-A	REL10950	40,000	4,430,963	910,384	1	3.39	1,083,668	*wrbA*	C→T	89.2	Genoscope
Ara+2	40K-B	REL10951	40,000	4,429,691	910,170	0	Inf.	-	-	-	86.8	Genoscope
Ara+4	40K-A	REL10956	40,000	4,441,213	910,966	3	1.57	420,328	*cyoB*	A(C	88.0	Genoscope
								2,772,320	*iap*	A(C		

								3,061,109	*ECB_02854*	G(A		
Ara+4	40K-B	REL10957	40,000	4,441,020	910,968	2	1.99	420,328	cyoB	A(C	95.2	Genoscope
								2,772,320	iap	A(C		
Ara+5	40K-A	REL10982	40,000	4,443,137	910,621	5	1.89	122,591	*ampE*	T→A	57.4	Genoscope
								212,865	*ldcC*	T→C		
								1,317,194	*trpC*	G→A		
								2,009,188	*yoeF*	G→T		
								2,251,393	*napA*	G→A		
Ara+5	40K-B	REL10983	40,000	4,443,794	910,724	5	1.83	122,591	*ampE*	T→A	66.5	Genoscope
								212,865	*ldcC*	T→C		
								1,317,194	*trpC*	G→A		
								2,009,188	*yoeF*	G→T		
								2,251,393	*napA*	G→A		

[a]Inf., indicates an infinite ratio between dN and dS because the denominator was zero.

[b]Genome position of synonymous substitutions relative to the ancestral strain, REL606 [GenBank:NC_012967.1].

[c]Facilities are KRIBB, Korea Research Institute of Bioscience and Biotechnology, Daejeon, ROK; MSU, Michigan State University, East Lansing, MI, USA; and Genoscope, Évry, FRA.

[d]Jeong, H., V. Barbe, C. H. Lee, D. Vallenet, D. S. Yu *et al*., 2009 Genome sequences of *Escherichia coli* B strains REL606 and BL21(DE3). J. Mol. Biol. **394:** 644–652.

[e]Barrick, J. E., D. S. Yu, S. H. Yoon, H. Jeong, T. K. Oh *et al.*, 2009 Genome evolution and adaptation in a long-term experiment with *Escherichia coli*. Nature **461:** 1243–1247.
